# Endoscopic lateral neck dissection *via* the breast and transoral approaches for papillary thyroid carcinoma: A preliminary report

**DOI:** 10.3389/fsurg.2022.997819

**Published:** 2022-11-01

**Authors:** Penghao Kuang, Yuanyuan Wang, Guoyang Wu, Yezhe Luo, Jinbo Fu, Wei Yan, Suqiong Lin, Xiaoquan Hong, Fusheng Lin, Ende Lin, Yilong Fu

**Affiliations:** ^1^ Department of General Surgery, Zhongshan Hospital, Xiamen University, Xiamen, China; ^2^ Department of Thyroid Surgery, Zhengzhou University First Affiliated Hospital, Zhengzhou, China

**Keywords:** thyroid, papillary thyroid carcinoma, endoscopic lateral neck dissection, lymph node metastasis, thyroid cancer

## Abstract

**Purpose:**

Complete lymph node dissection is essential for the management of papillary thyroid carcinoma (PTC) with lymph node metastasis (LNM). This work aimed to describe the feasibility of endoscopic lateral neck dissection *via* the breast and transoral approach (ELNDBTOA) in PTC patients and the necessity of the addition of the transoral approach.

**Methods:**

We included 13 patients with PTC and suspected lateral LNM who underwent ELNDBTOA at the Zhongshan Hospital, Xiamen University. Total thyroidectomy, ipsilateral central lymph node dissection, and selective neck dissection (levels IIA, IIB, III, and IV) were performed endoscopically *via* the breast approach. Residual lymph nodes were further dissected *via* the transoral approach.

**Results:**

The mean operation time was 362.1 ± 73.5 min. In the lateral neck compartments, the mean number of retrieved lymph nodes was 36.6 ± 23.8, and the mean number of positive lymph nodes was 6.8 ± 4.7. In further dissection via the transoral approach, lymph nodes in the lateral neck compartment were obtained in nine patients (9/13, 69.2%), and three patients (3/13, 23.1%) had confirmed lateral neck metastases. Transient hypocalcemia occurred in two patients (2/13, 15.4%), and three patients (3/13, 23.1%) developed transient skin numbness in the mandibular area. No other major complications were observed. There was no evidence of local recurrence or distant metastasis during the follow-up period (range, 24–87 months). All patients were satisfied with the good cosmetic outcome.

**Conclusion:**

ELNDBTOA is an option with proven feasibility for select PTC patients with LNM, and the addition of the transoral approach is necessary to ensure complete dissection.

## Introduction

Papillary thyroid carcinoma (PTC), the most common malignant thyroid tumor (1), is characterized by a high rate of lymph node metastasis (LNM), mostly in the central lymph nodes and sometimes in the lateral lymph nodes. LNM has a significant impact on PTC prognosis (2). Therefore, ensuring complete and legitimate dissection of the lymph nodes is crucial for the surgical management of PTC with LNM. Improvements in endoscopic thyroidectomy have allowed surgeons more options for lateral neck dissection. Tan et al (3). applied the transoral endoscopic technique during lateral neck dissection for selected patients. Other studies have described the chest-breast (4), chest (5), and breast approaches (6), and others have verified the safety of robot-assisted (7), needle-assisted (8), and video-assisted endoscopic lateral neck dissections (9). However, endoscopic lateral neck dissection *via* the breast and transoral approach (ELNDBTOA) has not been reported. In the breast approach, obstruction by the sternum and clavicles may cause a blind area in the surgical visual field, leading to incomplete dissection. We believe that the addition of the transoral approach can overcome this limitation. Herein, we report our experience using ELNDBTOA in the management of 13 PTC patients analyze the feasibility and of this approach and the necessity of the addition of the transoral approach.

## Materials and methods

### Ethical approval

This study was approved by the Medical Ethics Committee of Zhongshan Hospital, Xiamen University, Xiamen, Fujian, China. Written informed consent was obtained from all individual patients included in this study.

### Patients

Thirteen patients underwent ELNDBTOA by the same surgeon in our department between February 2015 and May 2020. PTC was diagnosed using ultrasonography-guided fine needle aspiration in all patients. Ultrasonography and/or computed tomography findings led to the suspicion of lateral LNM, which was confirmed using fine needle aspiration, *BRAF* mutation analysis, or washout thyroglobulin test.

The inclusion criteria for ELNDBTOA were as follows: (1) PTC with LNM; (2) the maximum diameter of primary tumor less than 4 cm; (3) metastatic lymph nodes not blended with each other or fixed in the neck; (4) no invasion of the neighboring structures, such as the esophagus, trachea, or recurrent laryngeal nerve; (5) no distant metastasis; (6) need for a cosmetic outcome; and (7) provision of informed consent for ELNDBTOA. The following exclusion criteria were used: (1) neck surgical or irradiation history, (2) level I or level V LNM, (3) hyperthyroidism or Hashimoto's thyroiditis, and (4) intolerance to general anesthesia.

Patient demographics, outcomes, and complications were collected retrospectively.

### Surgical procedures

#### Total thyroidectomy and central lymph node dissection *via* the breast approach

**Figure 1:**
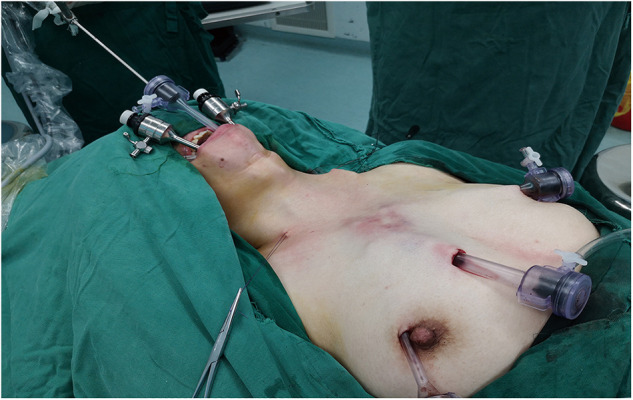
The distribution of trocars.

The preoperative preparations and major procedures of total thyroidectomy and central lymph node dissection *via* the breast approach have been described in detail previously (10). The distribution of trocars is shown in Figure 1.

#### Lateral neck dissection *via* the breast approach and transoral approach

**Figure 2:**
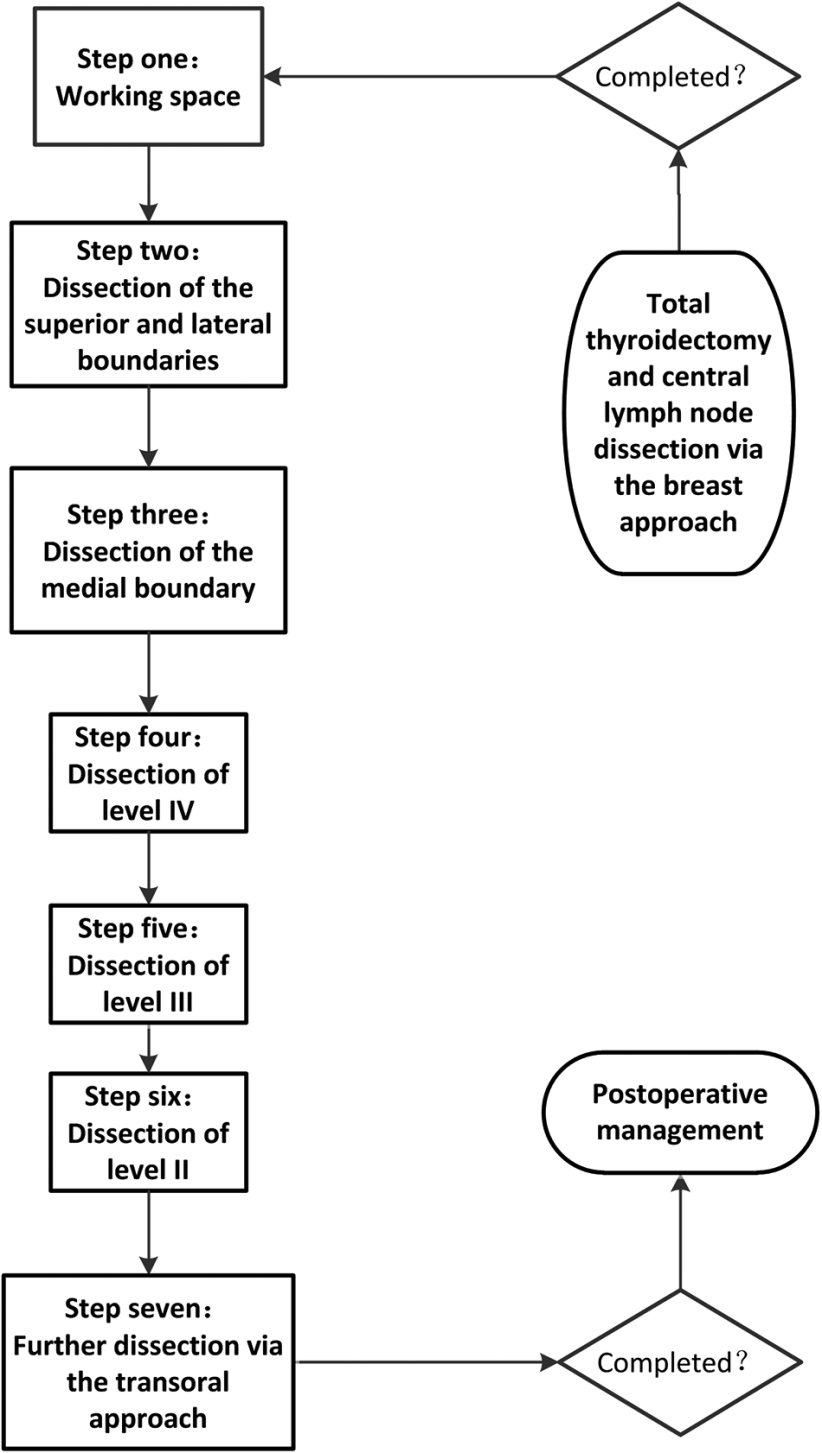
Seven steps of ELNDBTOA.

**Figure 3:**
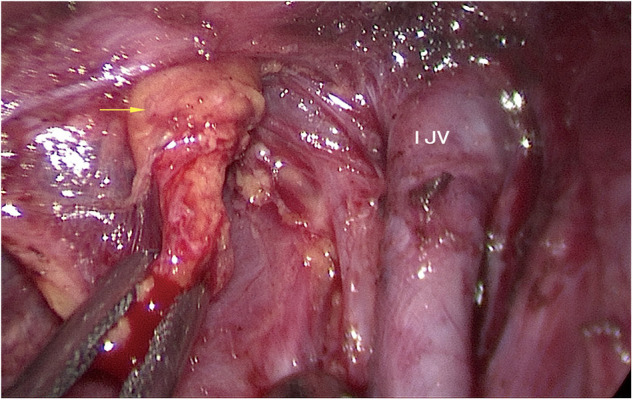
The left side residual lymph nodes (yellow arrow). IJV: internal jugular vein.

ELNDBTOA was performed using the following seven steps (Figure 2), with intraoperative neuromonitoring:

1) Working space: When total thyroidectomy and central lymph node dissection *via* the breast approach completed, we expanded the working space of the affected side to reach the hyoid level and the lateral margin of the sternocleidomastoid muscle (SCM).
2) Determination of the superior and lateral boundaries: The submandibular gland and posterior belly of the digastric muscle were exposed. The space between the sternal and clavicular heads of the SCM was split to expose the lateral cervical compartment, reaching the posterior margin of the SCM as the lateral boundary. Care was taken to identify and expose the accessory nerve without injuring it. Then, the tissue on the surface of the accessory nerve was dissected, and the superior boundary of level II was determined by cutting off the tissue of the lower edge of the digastric muscle.
3) Dissection of the medial boundary: The omohyoid muscle was exposed and preserved. The tissue of the carotid triangle region, the lymph nodes between the SCM and sternohyoid muscle, and the tissue on the surface of the internal jugular vein were dissected from top to bottom along the lateral margin of the strap muscles. Care was taken to separate the jugular vein angle, and the thoracic duct (left side) or the lymphatic trunk (right side) was clamped with a 5-mm hemolock to prevent chyle leakage.
4) Dissection of level IV: The inferior boundary of the lateral cervical compartment was separated from the jugular vein angle to the posterior margin of the SCM. The level IV tissue was lifted upward; then, level IV was dissected carefully without damaging the transverse cervical artery.
5) Dissection of level III: The lymph nodes of level III were dissected along the internal jugular vein from bottom to top in front of the prevertebral fascia. The lateral margin of level III was removed at the posterior margin of the SCM.
6) Dissection of level II: The lymph nodes of level II were dissected upward until the superior boundary, which had been separated in step 2, was reached. The accessory nerve was properly protected during this process.
7) Further neck dissection *via* the transoral approach and placement of the drainage tube: When these procedures were completed, three 5-mm incisions were made in the oral vestibule (Figure 1). A 5-mm, 30-degree laparoscope was used for observing the operative region. The residual lymph nodes located in levels VI and IV and between the SCM and sternohyoid muscle were dissected and removed carefully through the transoral approach (Figure 3). A total of 1,000 ml of warm saline was used for flushing the surgical region to check for active bleeding, and all incisions were sutured using absorbable sutures. Two drainage tubes were placed: one in the central cervical compartment and the other in the lateral cervical compartment.

#### Postoperative management and follow-up

**Figure 4:**
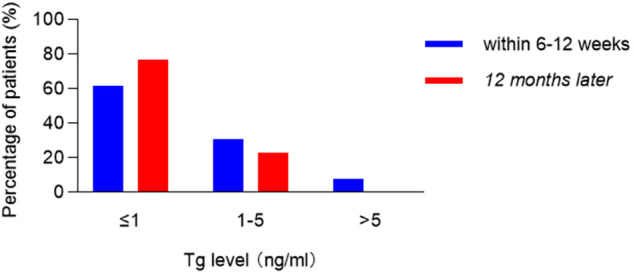
The percentage of patients with different postoperative thyroglobulin levels.

All patients started a semi-liquid diet after a postoperative 6-hour fast. Parathyroid hormone and serum calcium levels (Figure 4) were routinely checked on postoperative day 2. Complications were monitored for quick identification and prompt management. When the intraoperative recurrent laryngeal nerve signal decreased by more than 50%, intraoperative loss of signal was considered, and the fiberoptic nasopharyngoscope was performed on the first day postoperatively to determine whether there was vocal cord paralysis. If postoperative vocal cord paralysis occurs, according to the duration, temporary recurrent laryngeal nerve injury was diagnosed if it recovers within 6 months, and permanent recurrent laryngeal nerve injury was diagnosed when it lasts more than 6 months. Mild symptoms of hypoparathyroidism may manifest as numbness in the lips, face, and limbs, and twitching of the limbs, while severe symptoms may manifest as spasms of the larynx and diaphragm, causing difficulty in breathing. If the patient was diagnosed with hypoparathyroidism after surgery, symptomatic treatment should be given. If the patient recovers within 6 months, it is temporary hypoparathyroidism. Permanent hypoparathyroidism may be considered if there is no recovery after more than 6 months. The drainage tubes were removed when the volume was less than 15 ml/day. All the patients received thyroid-stimulating hormone suppression therapy, and they were encouraged to undergo radioactive iodine therapy in the oncology department 1–3 months later.

The first follow-up was completed 1 month after operation, and patients were followed up every 6 months thereafter. During the follow-up period, check for tumor recurrence and metastasis (imaging, thyroglobulin, thyroid function, etc.) and observe whether there are appearance deformities, sensory and movement abnormalities in the chest, neck and oral cavity.

## Results

ELNDBTOA was performed successfully with a successful cosmetic outcome in 13 patients (12 women and 1 man; age range, 22–53 years). The average tumor size was 2.1 cm ± 1.1 cm. The mean operation time was 362.1 ± 73.5 min. The mean intraoperative blood loss was 21.5 ± 13.4 ml. In the lateral neck compartments, the mean number of retrieved lymph nodes was 36.6 ± 23.8, and the mean number of positive lymph nodes was 6.8 ± 4.7. In further dissection via the transoral approach, lymph nodes in the lateral neck compartment were obtained in nine patients (9/13, 69.2%), and three patients (3/13, 23.1%) had confirmed LNM. The average length of hospital stay was 5.0 ± 1.2 days. All patients had normal parathyroid hormone and serum calcium levels on postoperative day 2. The clinical characteristics of the patients are shown in Table 1.

**Table 1:** The clinical characteristics of the patients (*n* = 13).

Case	Age (years)	Sex	Tumour size (mm)	Operation time (min)	TNM stage	Positive/retrieved number of lymph node (breast approach)		Positive/retrieved number of lymph node (transoral approach)	
						CNC	LNC	CNC	LNC
1	40	F	33	377	T2N1_b_M0	5/11	2/19	0/0	0/0
2	31	F	24	331	T2N1_b_M0	0/0	5/31	0/0	0/0
3	22	F	39	477	T2N1_b_M0 (L)	6/8 (L)	7/14 (L)	0/0 (L)	2/2 (L)
					T1_a_N1_a_M0 (R)	1/4 (R)	0/8 (R)	0/1 (R)	0/3 (R)
4	46	F	10	268	T1_b_N1_b_M0	1/4	2/20	0/1	0/2
5	34	F	26	327	T2N1_b_M0	1/1	8/14	0/0	0/0
6	33	F	8	423	T3N1_b_M0	2/3	4/40	0/0	0/3
7	53	F	29	272	T1_a_N1_b_M0	1/2	6/23	0/0	0/0
8	30	F	22	497	T3N1_b_M0 (L)	1/1 (L)	15/51 (L)	0/0 (L)	0/0 (L)
					T1_a_N1_b_M0 (R)	2/9 (R)	3/49 (R)	0/0 (R)	1/4 (R)
9	25	F	8	318	T3N1_b_M0	3/6	5/33	0/2	0/1
10	37	M	6	335	T3N1_b_M0	3/6	5/21	0/1	0/4
11	34	F	31	360	T1_b_N1_b_M0	9/12	10/26	0/0	1/4
12	52	F	22	423	T3N1_b_M0	4/9	2/33	0/0	0/9
13	25	F	18	299	T1_b_N1_b_M0	6/16	10/61	0/0	0/1

CNC, central neck compartment; LNC, lateral neck compartment; L, left side; R, right side.

None of the patients developed major complications (e.g., postoperative bleeding, infection, chyle leakage, and vocal cord paralysis). However, two patients (2/13, 15.4%) had transient hypocalcemia, which was reversed within 2 months, and three patients (3/13, 23.1%) developed transient skin numbness in the mandibular area and recovered within 2 weeks. The median follow-up period was 59 months (range, 24–87 months). There was no evidence of local recurrence or distant metastases. Postoperative thyroglobulin level with levothyroxine suppression was low (<1 ng/ml) in most patients (76.9%) after 12 months. The incisions in the oral cavity and breast healed well in all the patients, and they were satisfied with the good cosmetic outcome.

## Discussion

Because LNM is related to tumor recurrence in PTC patients, lateral neck dissection is the preferred and most efficient curative option for PTC with LNM according to the current guidelines (11). However, a long cervical incision is required for open lateral neck dissection, which is a limitation with regard to cosmetic outcome and medical privacy. Therefore, endoscopic techniques for lateral neck dissection have been explored. Miccoli et al. introduced a video-assisted technique in 2008 (12). Zhang et al (9). later reported that there was no significant difference in the number of lymph nodes obtained between video-assisted and open lateral neck dissection (41.1 ± 12.9 vs. 43.8 ± 13.1, *p* = 0.3194). Yan et al (6). described lateral neck dissection *via* the breast approach, and the mean number of retrieved lymph nodes in the lateral neck compartment was 21.8 (range, 5–42). In the pilot report of the transoral approach, the mean number of retrieved lymph nodes in levels III and IV was 10.9 ± 2.8 (3). However, the breast approach may result in incomplete lymph node dissection. In this study, we aimed to analyze the feasibility and necessity of ELNDBTOA.

### Feasibility and necessity of this technique

The safety and efficacy of radical excision should be the principal areas of focus when considering surgical approaches rather than the cosmetic outcome. In this study, we described endoscopic lateral neck dissection (levels IIA, IIB, III, and IV) *via* the breast combined with the transoral approach in detail. All 13 patients underwent ELNDBTOA successfully without conversion to open surgery. In the lateral neck compartment, the mean number of retrieved lymph nodes was 36.6 ± 23.8, and three patients (3/13, 23.1%) had confirmed LNM. With regard to complications related to the additional transoral ports, three patients had transient skin numbness in the mandibular area, which resolved within 2 weeks. No other patients developed complications from the procedure. There was no tumor recurrence or metastasis during the follow-up period (range, 24–87 months). Hence, ELNDBTOA is considered a safe and effective technique that is worthy of promotion in well-selected patients.

The key to lateral neck dissection is complete resection, since incomplete resection can have serious consequences requiring further treatment. Some patients may need a second surgery or even multiple operations due to nonstandard and incomplete initial surgery. In this report, three patients (23.1%) had confirmed LNM diagnosed during further dissection using the transoral approach, highlighting the necessity of the addition of the transoral approach. This transoral approach is necessary because the surgical visual perspective of the breast approach is blocked by the sternal manubrium and clavicles, which may lead to blinding the area of the visual field, resulting in incomplete dissection in the central neck compartment, level IV, or lymph nodes between the SCM and sternohyoid muscle. In contrast, the transoral approach provides sufficient exposure to the surgical visual field in these areas. Hence, combining the transoral approach with the breast approach for lateral neck dissection is required.

### Surgical extent and patient selection

The optimal surgical extent of lateral neck dissection is still controversial, mainly in the dissection of level IIB and level V lymph nodes. Some authors who are opposed to routine dissection in level IIB and level V argue that the accessory nerve and C4 (the fourth branch of the cervical plexus) could be protected to a certain extent.

**Figure 5:**
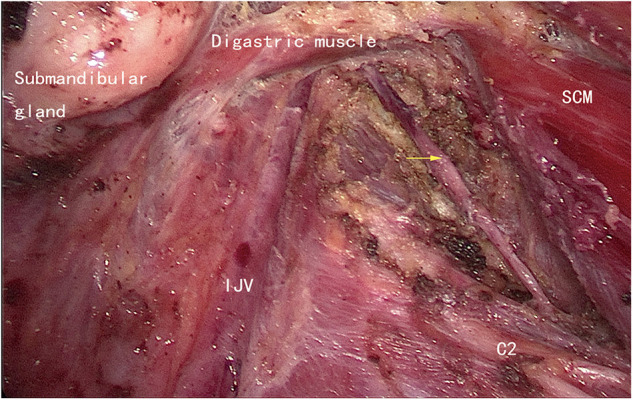
The superior boundary (left side). SCM, sternocleidomastoid muscle; IJV, internal jugular vein; C2, the second branch of the cervical plexus; yellow arrow: the accessory nerve.

**Figure 6:**
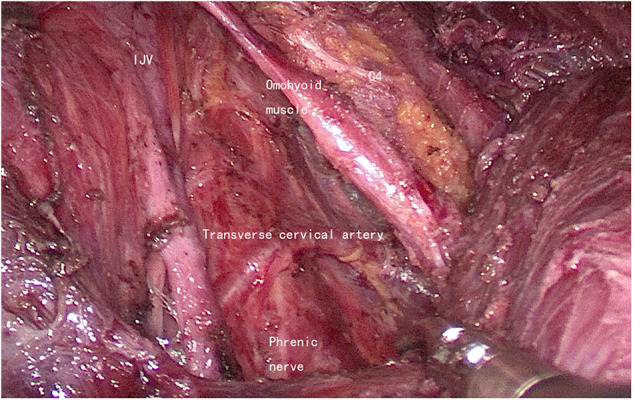
The inferior boundary (left side). IJV, internal jugular vein; C4, the fourth branch of the cervical plexus.

The boundaries for lateral neck dissection (level II, level III, and level IV) in this study were as follows: the inferior margin of the posterior belly of the digastric muscle as the superior boundary (Figure 5), the level of the clavicle as the inferior boundary (Figure 6), the medial margin of the common carotid artery as the medial boundary, and the posterior margin of the SCM as the lateral boundary. The reasons for the routine dissection of level IIB: level IIA and level IIB encompass a single piece of tissue, although they are anatomically bounded by the accessory nerve; protecting the accessory nerve would be difficult if a second operation is needed later. Patients with preoperative suspected metastases in level I or V should not undergo ELNDBTOA.

Based on our experience, we propose that ELNDBTOA should be performed in patients with a cosmetic requirement who are diagnosed with PTC and suspected LNM in the low position of level IV that may be blocked by the clavicle and sternum.

### The prevention of complications

There are several complications related to lateral neck dissection; therefore, we explored means to prevent these complications in patients undergoing ELNDBTOA.

Chyle leakage has been reported as a common complication of lateral neck dissection (occurring in 5.1% of total thyroidectomies with ipsilateral lateral neck dissection and 6.2% of total thyroidectomies with bilateral lateral neck dissection) (13) probably due to intraoperative injury to the thoracic duct or lymphatic trunk. None of our patients developed chyle leakage. To avoid chyle leakage, we recommend that no energy instruments be used, but a 5-mm hemolock should be used in the dangerous area between the transverse cervical vessels and the venous angle if a cord structure is seen. Chyle leakage should be carefully assessed at the end of surgery. If the thoracic duct or lymphatic trunk is injured, 6-0 Prolene can be used for suturing.

Severe intraoperative bleeding is a thyroid endoscopic surgical emergency, which may not only lead to conversion to open surgery but also cause CO_2_ embolism. All surgeries were performed endoscopically in this study, and no CO_2_ embolism occurred. Based on our previous experience with endoscopic thyroid surgery (14), we recommend that surgeons enhance their anatomical knowledge (e.g., of the external carotid vein, all branches of the internal jugular vein, common carotid artery, superior thyroid artery, and SCM perforator parts) and operate with great care. If severe intraoperative bleeding occurs, CO_2_ embolization should be suspected, identified, and treated promptly.

The accessory nerve is the boundary between level IIA and level IIB. Injury of this nerve leads to paralysis and atrophy of the trapezius, which will significantly affect the patient's postoperative quality of life. None of our patients had accessory nerve injury, and we suggest that the surgeon should make full use of the high resolution of endoscopy to reveal the anatomical structure, expose the accessory nerve, and avoid injury.

### Limitations

The mean operation time was 362.1 ± 73.5 min. This means that the patients were under general anesthesia for a long period. Therefore, surgeons should strengthen their proficiency to shorten the operation time. Moreover, the patient must undergo a rigorous anesthesia tolerance assessment before surgery. Because of the small sample size, a further study including a larger sample size and comparison with the breast approach is needed.

## Conclusion

In conclusion, ELNDBTOA is an option with proven feasibility for select PTC patients with LNM, and the addition of the transoral approach is necessary to ensure complete dissection.

## Data Availability

The original contributions presented in the study are included in the article, further inquiries can be directed to the corresponding author.

## References

[1] Cabanillas M McFadden DG Durante C. Thyroid cancer. Lancet. (2016) 388:2783–95. 10.1016/S0140-6736(16)30172-6 27240885

[2] Zaydfudim V Feurer ID Griffin MR Phay JE. The impact of lymph node involvement on survival in patients with papillary and follicular thyroid carcinoma. Surgery. (2008) 144:1070–7. 10.1016/j.surg.2008.08.034 19041020

[3] Tan Y Guo B Deng X Ding Z Wu B Niu Y Transoral endoscopic selective lateral neck dissection for papillary thyroid carcinoma: a pilot study. Surg Endosc. (2020) 34(12):5274–82. 10.1007/s00464-019-07314-8 31834511

[4] Guo Y Qu R Huo J Wang C Hu X Chen C Technique for endoscopic thyroidectomy with selective lateral neck dissection via a chest-breast approach. Surg Endosc. (2019) 33:1334–41. 10.1007/s00464-018-06608-7 30569419

[5] Lin P Liang F Cai Q Han P Chen R Xiao Z Comparative study of gasless endoscopic selective lateral neck dissection via the anterior chest approach versus conventional open surgery for papillary thyroid carcinoma. Surg Endosc. (2021) 35(2):693–701. 10.1007/s00464-020-07434-6 32076863

[6] Yan H Wang Y Wang P Xie Q Zhao Q. “Scarless” (in the neck) endoscopic thyroidectomy (SET) with ipsilateral levels II, III, and IV dissection via breast approach for papillary thyroid carcinoma: a preliminary report. Surg Endosc. (2015) 29:2158–63. 10.1007/s00464-014-3911-1 25427410

[7] Paek SH Lee HA Kwon H Kang K Park S. Comparison of robot-assisted modified radical neck dissection using a bilateral axillary breast approach with a conventional open procedure after propensity score matching. Surg Endosc. (2019) 34:622–7. 10.1007/s00464-019-06808-9 31065778

[8] Wang B Weng YJ Wang SS Zhao W Yan S Zhang L Feasibility and safety of needle-assisted endoscopic thyroidectomy with lateral neck dissection for papillary thyroid carcinoma: a preliminary experience. Head Neck. (2019) 41(7):2367–75. 10.1002/hed.25705 30775820

[9] Zhang D Gao L Xie L He G Chen J Fang L Comparison between video-assisted and open lateral neck dissection for papillary thyroid carcinoma with lateral neck lymph node metastasis: a prospective randomized study. J Laparoendosc Adv Surg Tech A. (2017) 27:1151–7. 10.1089/lap.2016.0650 28488911

[10] Wu G Fu J Lin F Luo Y Lin E Yan W. Endoscopic central lymph node dissection via breast combined with oral approach for papillary thyroid carcinoma: a preliminary study. World J Surg. (2017) 41:2280–2. 10.1007/s00268-017-4015-6 28417186

[11] Haugen B Alexander E Bible K Doherty G Mandel S Nikiforov Y American thyroid association management guidelines for adult patients with thyroid nodules and differentiated thyroid cancer: the American thyroid association guidelines task force on thyroid nodules and differentiated thyroid cancer. Thyroid. (2015) 2016(26):1–133. 10.1089/thy.2015.0020 PMC4739132 26462967

[12] Miccoli P Materazzi G Berti P. Minimally invasive video-assisted lateral lymphadenectomy: a proposal. Surg Endosc. (2008) 22:1131–4. 10.1007/s00464-007-9564-6 17721805

[13] Lee Y Nam K Chung W Chang H Park C. Postoperative complications of thyroid cancer in a single center experience. J Korean Med Sci. (2010) 25:541–5. 10.3346/jkms.2010.25.4.541 20357995 PMC2844597

[14] Fu J Luo Y Chen Q Lin F Hong X Kuang P Transoral endoscopic thyroidectomy: review of 81 cases in a single institute. J Laparoendosc Adv Surg Tech A. (2018) 28:286–91. 10.1089/lap.2017.0435 29297741

